# Cost-effectiveness analysis of parenting interventions for the prevention of behaviour problems in children

**DOI:** 10.1371/journal.pone.0225503

**Published:** 2019-12-02

**Authors:** Camilla Nystrand, Inna Feldman, Pia Enebrink, Filipa Sampaio

**Affiliations:** 1 Department of Public Health and Caring Sciences, Uppsala University, Uppsala, Sweden; 2 Department of Clinical Neuroscience, Karolinska Institute, Stockholm, Sweden; University College Dublin, IRELAND

## Abstract

**Background:**

Behavior problems are common among children and place a high disease and financial burden on individuals and society. Parenting interventions are commonly used to prevent such problems, but little is known about their possible longer-term economic benefits. This study modelled the longer-term cost-effectiveness of five parenting interventions delivered in a Swedish context: Comet, Connect, the Incredible Years (IY), COPE, bibliotherapy, and a waitlist control, for the prevention of persistent behavior problems.

**Methods:**

A decision analytic model was developed and used to forecast the cost per averted disability-adjusted life-year (DALY) by each parenting intervention and the waitlist control, for children aged 5–12 years. Age-specific cohorts were modelled until the age of 18. Educational and health care sector costs related to behavior problems were included. Active interventions were compared to the waitlist control as well as to each other.

**Results:**

Intervention costs ranged between US$ 14 (bibliotherapy) to US$ 1,300 (IY) per child, with effects of up to 0.23 averted DALYs per child (IY). All parenting interventions were cost-effective at a threshold of US$ 15,000 per DALY in relation to the waitlist control. COPE and bibliotherapy strongly dominated the other options, and an additional US$ 2,629 would have to be invested in COPE to avert one extra DALY, in comparison to bibliotherapy.

**Conclusions:**

Parenting interventions are cost-effective in the longer run in comparison to a waitlist control. Bibliotherapy or COPE are the most efficient options when comparing interventions to one another. Optimal decision for investment should to be based on budget considerations and priority settings.

## Introduction

The European Union (EU) [1], as well as the Lancet Psychiatry Commission [2], emphasize the increasing need to focus on sustainable mental health related promotion, disease prevention and its economic benefits. A mean towards this end is to identify effective interventions and, against competing demands, allocate scarce resources to produce the best *value-for-money*. In Sweden, it is suggested that an increasing amount of resources ought to be directed towards psychiatric services [3], especially preventive strategies. However, for sustainable health promotion, a longer-term efficiency perspective is needed [4].

Mental health related problems may arise in childhood and early adolescence, with as much as half of all cases developing to chronic disorders starting before the age of 14 [5]. Among those peaking in the early ages of life are externalizing behaviour problems [6], such as inattention, hyperactivity, oppositional or deviant behaviour. Somewhere between 5–20% of children and young adolescents experience conduct problems (CP) [7] or attention-deficit and hyperactivity problems (ADHP) [8]. Pathological behavior’s such as these often co-exist [8], especially in children with an early age of onset [9]. Externalizing problems augment the risk of several consequences arising later in life, including school drop-out, alcohol and drug abuse, and antisocial and criminal behaviour [10, 11]. This places a high disease burden on individuals, but also a financial burden on them, their families and society. Scott et al. [12] estimated that children with CP followed until the age of 28 cost the society three and a half times higher than children without problems.

Reasonably, due to the high risks in early childhood for prolonged problems, coexistence of problems and related risks in adulthood, early interventions are key. As children spend a large amount of time in their family environment, it is an important platform for prevention [13]. One such way is by strengthening parenting management skills and the parent-child relationship, which has shown to reduce child behaviour problems [14, 15] with effects persisting at least up to three years post intervention [16]. In Sweden, parenting interventions are offered by most local authorities to parents that experience difficulties in managing their children [17]. For children whose parents seek help through child and adolescent psychiatric services, a recommended first line approach is in fact parenting programs [18].

The cost of delivering parenting interventions is relatively low, with group-based interventions costing on average per child US$ 135 for 1-2-3 Magic parenting program [19] and up to US$ 1,890 for the Incredible Years (IY) [20]. We also know from economic evaluations that most parenting interventions delivered as indicated prevention are cost-effective and that investments in such may improve health-related quality of life [19, 21–23]. Attempts have previously been made to forecast the longer-term economic gains of parenting interventions, such as the IY program [20] and Triple P [24, 25]. Results show that they may generate future health, economic and societal returns.

A randomized controlled trial (RCT) in Sweden investigating the impact of several parenting interventions shows that almost all are effective at reducing externalizing problems in comparison to a waitlist control [26]. A separate study looking at the economic credentials of these interventions concludes that the interventions significantly reduce externalizing problems at modest costs [27]. However, the study is limited in that it only considers post-test effects, did not include resource use, and did not use a multi-attribute utility instrument (MAUI) to estimate health related quality of life. This limits the possibility of drawing conclusions as there is no established willingness-to-pay for a reduction in symptom levels of behaviour problems. In relation to these limitations, we intend to build on the effectiveness and cost-effectiveness of the trial to determine whether these programs are cost-effective in a longer-run perspective, including a longer-term follow-up of effects, resource use and DALYs, to inform decision makers within public health.

### Aim of the Study

The aim was to forecast the longer-term cost-effectiveness of five parenting interventions delivered in a Swedish context; COPE, Connect, Comet and the IY, a self-help book, and a waitlist control, for the prevention of persistent externalizing behaviour problems—CP, ADHP, and co-existing ADHP/CP.

## Methods

### Economic evaluation framework

A long-term economic evaluation was conducted, comparing five different types of parenting interventions to a waitlist control, for the prevention of persistent externalizing behaviours in 5–12 year old children. The evaluation forecasted the potential benefits and costs to the children until end of childhood (18 years of age) through a health state-transition model. Intervention effects and costs, as well as some epidemiological data were derived from the *original trial* [26]. Secondary epidemiological data was collected from the global burden of disease (GBD) [28], and costs related to externalizing behaviours were estimated from the published literature. Costs and health outcomes were discounted at a rate of 3% per annum with respect to the reference year 2015 in the base case analysis [29]. Health outcomes were estimated as disability-adjusted life-years (DALYs). All prices were converted to 2015 US$ using purchasing power parities. Model parameters are found in Table 1 and sources, specific estimations, analyses and assumptions are further explained in a technical report [30]. Modelling results are presented as average DALYs and average total costs (for the intervention and costs related to the problems) per individual, for each arm. Incremental cost-effectiveness ratios (ICERs) were estimated for each active intervention in comparison to the control, producing estimates of the net costs per averted DALY. Furthermore, interventions were compared to each other and ranked according to the principle of extended dominance [31]. An ICER was calculated for the two best ranked alternatives. A willingness-to-pay (WTP) threshold of US$ 80,000 [32] per averted DALY was used to assess cost-effectiveness, as this level represents what is normally accepted for reimbursement of new pharmaceuticals in Sweden.

**Table 1:** The features of the interventions evaluated within the trial: COPE, Connect, Comet, the Incredible Years and bibliotherapy.

Intervention	Age range	Sessions/frequency/participants	Components	Theory
Cope	3–12 years	10 2–2.5-hour sessions weekly Maximum 25 parents/group	Group discussions Modeling Role play Home work Self-monitoring	Social learning theory, some principles on cognitive and social psychological models on attitude change and family systems theory.
Connect	9–16 years	10 1-hour sessions weekly 12–14 parents	Teaching Role playing Hand-outs to parents	Attachment theory; systemtic theories; relational theories
Comet	3–12 years	11 2.5-hour sessions weekly 10–12 parents	Teaching Role playing Home work Video vignettes Hand-outs to parents One individual meeting	Based on Webster-Stratton’s and Patterson’s [38] and Barkley’s [39] parent management models; cognitive behavioural therapy
The Incredible Years	3–8 years	12 2–2.5-hour sessions weekly 10–14 parents	Teaching Role plays Group discussions Weekly homework Videotaped modeling Phone calls, make-up calls Buddy calls	Cognitive social leatning theory, Pattersons’s [38] coercion model; Bandura [40] notions of modeliung and self-efficacy; Piaget and Inhelder [41] developmental interactive learning methods
Bibliotherapy	2–12 years	-	-	Developed based on the program Comet

### Interventions evaluated

This economic evaluation included the following arms; the parenting programs COPE [33], Connect [34], Comet [35] and the Incredible Years (IY) [36], a self-help parenting management book (bibliotherapy) called “Five times more love” [37], and a waitlist control. COPE, Comet and the IY aim to improve children’s emotional skills and regulation by promoting cooperative behaviour, ignoring inappropriate behaviours, and using limit setting, rules and routines. Connect, on the other hand, builds upon the parent-child relationship and focuses on creating empathy, adjusting child behaviour and changing emotional responses. The self-help book was given to parents to improve parenting strategies on their own, and is based on the same principles as Comet. More detailed information regarding the different interventions can be found in Table 1.

### Study population

The *original trial* was implemented in a non-clinical setting through local communities in Sweden, which before the start of the trial offered at least two of the four parenting programs. Hence, effectiveness of the interventions was evaluated as if delivered in a real life setting. Interventions targeted parents of 3–12 year old children who experienced externalizing problems. A total of 1,104 parents were eligible and randomized in the trial. For children 3–8, parents were randomized to Comet (n = 207), COPE (n = 202) and the IY (n = 122). For children aged 9–12, randomization was done between Comet, COPE and Connect (n = 218). For bibliotherapy (n = 196) and the control group (n = 159), no age-restrictions were made. The IY had lower number of parents attending the intervention due to organizational problems and the geographical location of the sites offering the intervention. In addition to screening positive for externalizing problems, 48% of parents reported that their child had an ADHD diagnosis. No other diagnoses comprised more than 3%. Almost half (45.5%) of the parents had university level education, while 9% had only completed compulsory school education. Around 6% of parents were in the lowest income bracket (roughly USD$ 0–1000 per month). 89% of parents were born in a Scandinavian country. A majority of the sample of children were male. Further information regarding the randomization process, sample and attrition details and analysis can be found in the *original trial* [26].

### Data inputs for the model

#### Retrieved from the trial–epidemiology, effects and costs

The trial was approved by an ethics committee (Ethical approval number: DNR 2009/254) and all the data outlined in this section was collected from the same trial. Parent reported data were collected at baseline, four months after baseline (post-test) and two years after the intervention (follow-up), which included measures of child externalizing problems. The waitlist control was only held for four months. Therefore, the follow-up data only provided an indication of the stability of the effects of each intervention following the post-test measurement, without a comparison to the control. We used data from children between the ages 5–12, and conservatively excluded 3–4 year olds from the analyses due to data regarding resource use being unavailable for children with behavioural problems below the age of five. Hence, 160 children were excluded from the original trial sample and analyses were based on intention-to-treat with data from the randomized 944 individuals.

Health outcomes were related to parent-reported ADHP and CP. The Eyberg Child Behavior Inventory (ECBI) [42] was used to measure intensity and problems related to CP, while two subscales from the Swanson, Nolan and Pelham Scale (SNAP-IV) [43] were used to rate ADHP. Although not used as instruments for establishing diagnoses, their available cut-offs values were used as a proxy for clinical levels of either conduct -or hyperactivity/impulsivity problems, which has been used previously [44, 45]. We used the Clinically Significant Reliable Change Index (CS/RCI) [46] to estimate the amount of “recovered” cases in each trial arm, a method also used in the short-term cost-effectiveness study [27]. These were individuals (cases) remitted from any or both of the clinical levels of the problems, hence individuals were prevented from persisting with problems. Post-test rates were re-estimated to monthly rates and thereafter extrapolated to one-year probabilities as yearly cycle transitions were used in the state-transition model. The effects (proportion of recovered cases) are reported in Table 2. Using the CS/RCI method, we also estimated incidence and remission probabilities based on transitioning from clinical to non-clinical levels (recovered cases) for individuals in the control group, between baseline and post-test. These probabilities were also extrapolated to one-year probabilities. Incidence and remission probabilities were used in the decision-analytic model to inform yearly transition probabilities between health states.

**Table 2:** Parameters used in the decision analytic model.

Parameter		Value (95% CI or uncertainty range)						Uncertainty distribution	Source
**Epidemiological inputs (probabilities)**ᵃ				** **	** **	** **	** **	** **	** **
	All-cause mortality	0.00013							[47]
	** **	ADHP		CP		Comorbid ADHP/CP			
	Prevalence	0.002 (0.000–0.127)		0.460 (0.365–0.559)		0.191 (0.124–0.276)		Beta	Estimation based on trial and epidemiological data [26, 48]
	Incidence	0.000 (0.000–0.013)		0.004 (0.000–0.019)		0.008 (0.002–0.023)		Beta	
	Remission	0.105 (0.003–0.461)		0.133 (0.063–0.238)		0.191 (0.110–0.300)		Beta	
	Case fatality	0ᵇ		0ᵇ		0ᵇ			
**Effects (proportion of recovered cases)**		Post-test^c^	Two-year follow-up^d^	Post-test^c^	Two-year follow-up^d^	Post-test^c^	Two-year follow-up^d^		
	Comet	0.00 (0.00–0.04)	0.00 (0.00–0.04)	0.25 (0.17–0.33)*	0.23 (0.16–0.31)*	0.04 (0.02–0.08)	0.03 (0.01–0.07)	Beta	[26, 49]
	Connect	0.04 (0.01–0.10)	0.04 (0.01–0.10)	0.11 (0.06–0.18)	0.19 (0.12–0.27)*	0.03 (0.01–0.06)	0.03 (0.01–0.06)	Beta	
	Incredible Years	0.00 (0.00–0.11)	0.00 (0.00–0.11)	0.27 (0.16–0.32)*	0.37 (0.25–0.51)*	0.04 (0.01–0.11)	0.03 (0.01–0.09)	Beta	
	COPE	0.07 (0.03–0.14)	0.06 (0.03–0.14)	0.23 (0.16–0.32)*	0.31 (0.24–0.40)*	0.05 (0.02–0.09)*	0.06 (0.03–0.11)	Beta	
	Book	0.03 (0.01–0.10)	0.05 (0.01–0.10)	0.18 (0.04–0.10)*	0.22 (0.15–0.31)*	0.01 (0.00–0.03)	0.04 (0.01–0.08)	Beta	
	Waitlist	0.01 (0.00–0.05)		0.07 (0.03–0.13)		0.02 (0.00–0.05)		Beta	
Costs related to problem states									
	Health care	2,472 (899–3,954)		412 (Range ± 20%)		412 (Range ± 20%)^e^		Gamma/Triangular	ADHP costs: [50–56] CP costs: [12, 20] ADHP/CP costs: [12, 20]
	Education			1,091 (Range ± 20%)				Triangular	

*Notes*. GBD = Global Burden of Disease; ADHP = Attention-Deficit/Hyperactivity problems; CP = Conduct problems; CI = Confidence interval.

ᵃ All epidemiological inputs (except for population) in the table are averages between ages 5–18. In the decision analytic model, specific age probabilities were used. These are based on the trial and national epidemiological data, which is explained further in the technical report [30].

ᵇ As per the 2015 GBD study, case fatality was zero.

^c^ Effects are based on the post-test measurement (at four months post baseline) which were extrapolated to correspond to one year probabilities. All effects are reported as proportions of recovered cases, which were applied to the prevalence of each problem at the first (for post-test) and second cycle (for the two-year follow-up). Difference between intervention and control group effects were measured with chi-squared tests.

^d^ The effect is based on the two-year follow up. Because of the lack of a control group after the post-test time point, the probability estimate at post-test from the waitlist control has been used at the two-year time point as well, assuming a linear probability of remission.

^e^ Due to the lack of cost data for the comorbid state ADHP/CP, it was conservatively assumed that the resource use by the these individuals mirrored the lowest cost estimated for either ADHP or CP, although clinically it may make more sense that the higher cost would be more likely to be incurred by these individuals.

* p< .05, two-tailed

Costs related to delivering the various interventions were based on the *original trial* and estimated in the short-term cost-effectiveness study [27]. In the base case analysis, intervention costs were calculated based on all randomized parents (n = 944). However, as cost per child would be higher the fewer the parents attending the interventions’, one sensitivity analysis explored the impact of modelling only parents who completed the interventions (or read the full book) (n = 546) [27]. Intervention costs can be found in Table 3.

**Table 3:** Intervention costs for the parenting interventions (US$ 2015).

			Comet	Connect	Incredible Years	COPE	Bibliotherapy
Training costs			** **	** **	** **	** **	** **
		Training course fee	2 564	1 456	641	815	-
		Number of training days	8	3	3	3	-
		Average allowance per training day/practitioner	165	165	165	165	-
		Average hotel cost/practitioner	533	162	140	34	-
		Average trip cost/practitioner	76	81	70	16	-
		Average travel allowance/practitioner	16	17	15	4	-
Average total training cost/practitioner			4 615	3 064	2 209	2 192	-
Subtotal training cost^a^			226 112	110 318	44 172	59 191	-
	20% of total training cost		**45 222**	**22 064**	**8 835**	**11 838**	-
Running costs:							
	Time per session (hour)^b^		2.75	2	2.75	2.75	-
	Set of sessions/program		11	10	12	10	-
	Number of programs run		38	29	18	20	-
	Number of practitioners		49	36	20	27	-
	Time per practitioner running sessions		1 045	435	648	500	-
	Cost/ two practitioners running sessions		56 632	19 645	26 825	29 806	-
	Time per practitioner preparing sessions		836	435	1 987	400	-
	Cost/ two practitioners preparing sessions		41 187	19 645	89 743	21 677	-
	Rent of the venue		14 615	5 069	6 923	7 692	-
	Cost of materials (curriculum + student material)						2 335
	Yearly license fee		16 225				
Subtotal running cost			118 643	49 842	126 580	65 049	2 335
Number of children ITT/Intervention completers			176/126	215/103	104/53	161/68	162/58
Total intervention cost			163 865	71 906	135 414	76 888	2 335
	Average total cost/child per ITT		931	334	1 302	478	14
	Average total cost/child per intervention completer		1 301	698	2 555	1 131	40

Notes. All information is based on project documentation from trial on the same interventions. Further information can be obtained in the previously

published studies [26, 27]. Costs are presented in 2015 US$.

^a^ Average training cost per practitioner multiplied by total number of leaders per program

^b^ These are averages times during the trial, which were somewhat higher than the stipulated times reported in Table 1.

### Retrieved from the literature–epidemiology, cost offsets and DALYs

We collected disease and Swedish specific epidemiological data for conduct disorder and ADHD, corresponding to prevalence and incidence, from the GBD [48]. We retrieved data for five-year age groups between the ages 1–19 and interpolated the data with the use of EpigearXL [57] to obtain age-specific estimates. Data on remission from the disorders were derived from the literature and average annual remission probabilities were estimated. In addition, we used data from the literature to estimate co-existence between ADHD/conduct disorder. This data were collected to inform transition probabilities between health states in the model. All data, as well as search results, data extraction and analyses of remission probabilities and co-existing ADHD/conduct disorder are presented and explained more in detail in a technical report [30]. All cause mortality was estimated from national statistics [47].

Costs related to ADHP, CP and comorbid ADHP/CP were based on cost of illness studies conducted in a European setting, assuming a potential similarity to the Swedish welfare system. An attempt was made to include the full societal impact of externalizing behaviour problems, however estimation of cost offsets were limited to the health care and educational sector due to lack of data for other sectors and payers. Costs were collected for a population of children aged 5–18 years old, as the model follows individuals between these ages. Due to a limited number of studies, average costs were estimated for this age range, hence no age-specific costs were used. Costs in the included studies were yearly costs per child, which were applied in every yearly cycle in the model until the child reached 18 years of age. A full description of the steps taken and assumptions made regarding the calculation of the costs is presented in the technical report [30]. No studies were found looking at the costs for co-existing ADHP/CP. Therefore, we took a conservative approach and used the lowest cost found for either ADHP or CP. Table 2 includes the cost inputs and their corresponding sources.

Averted DALYs were used as the primary health outcome (disability weights were connected to the prevalence of the problems in each “problem” health state) and retrieved from the GBD study [58]. Case fatality was not accounted for in the model due to the lack of mortality related to the problems, hence DALYs were equivalent to years lived with disability.

### Health state transition model

**Fig 1:**
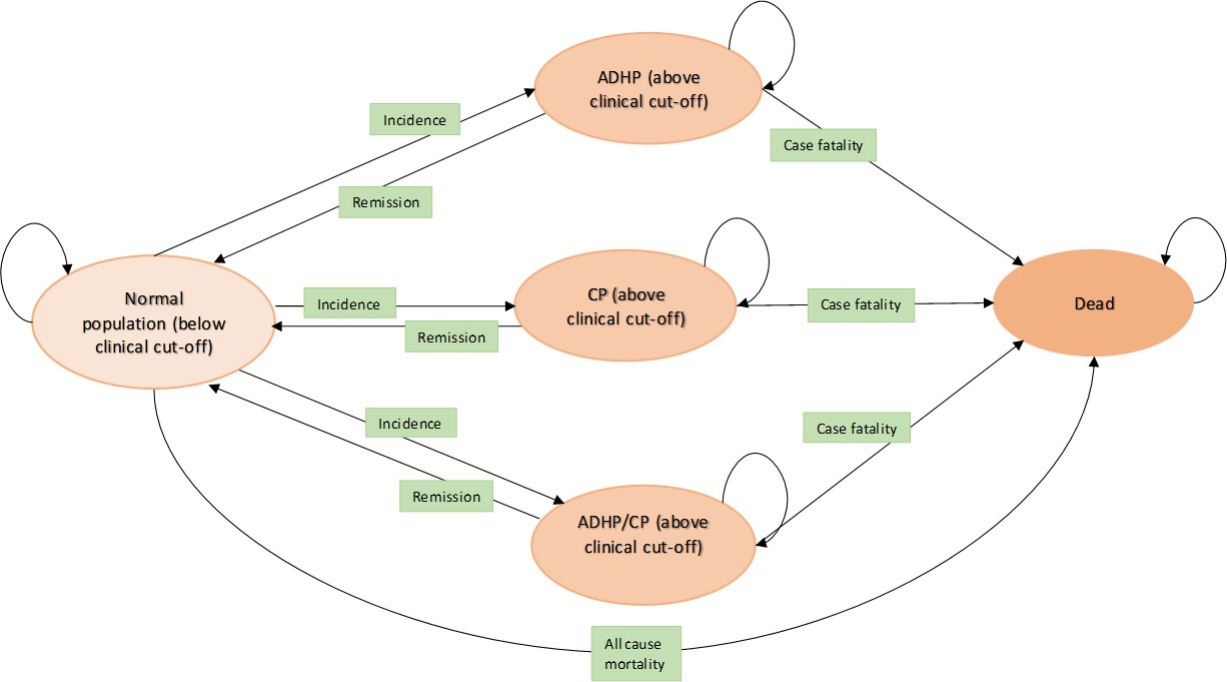
Conceptual model showing the transitions, due to epidemiological parameters, between the various health states. Notes: ADHP = Attention-Deficit/Hyperactivity problems; CP = Conduct problems.

A decision analytic multiple-cohort Markov model was developed to simulate the epidemiological pathways between non–chronic externalizing behaviour problems of an individual receiving any of the five parenting interventions or the waitlist control. The study modelled age-specific cohorts separately until the age of 18 and transitions between health states were done on an annual basis. Different cohorts were modelled for each intervention as interventions were provided to different age-groups, as explained previously. For instance, an individual aged between 5–8 years old was modelled up to the age of 18 for the IY program. The total amount of cycles depended on the starting age–from a minimum of six cycles (for a 12 year old) to 13 cycles (for a five year old). Results represent an average of the modelled aged. We modelled five different health states–sub-threshold population (below clinical cut-off), problems (clinical levels of ADHP, CP or co-existing ADHP/CP) and the absorbing dead state. At the starting cycle (year zero), the problem states included the proportion of individuals who had clinical levels of ADHP, CP and co-existing ADHD/CP estimated from the full trial sample, while the remaining population was in the sub-threshold state (see Fig 1 for a conceptual model of the health states and ways to transition between them). The probabilities of transitioning between states in consecutive cycles were estimated based on 1) data from the trial (incidence and remission as explained earlier) which was amended using the retrieved 2) data from the GBD and Swedish national statistics [47, 48]. Firstly, the trial sample was not large enough to estimate age-specific probabilities, hence we only had an age-average estimate of prevalence, incidence and remission. Secondly, as we modelled an individual until 18 years of age, additional data were needed for ages older than those included in the trial. As we modelled externalizing problems rather than disorders, solely using GBD data (which is reported for disorders only) would have underestimated prevalence and incidence, since the group of children already had externalizing problems at start. We therefore used information from both the trial and the GBD to calculate transition probabilities. How this was done is explained further in a technical report [30].

### Uncertainty and Sensitivity analyses

A probabilistic sensitivity analysis was conducted to account for uncertainty around model parameters, included in Table 1. We used Microsoft Excel 2016 to run Monte Carlo simulations with 1000 iterations. Univariate sensitivity analyses for certain parameters were run to estimate the impact of: (1) including only the "paying agency” perspective (hence only using educational sector costs), (2) using both “recovered” and “improved” cases in the estimation of effects, (3) doubling the intervention costs (4) using only post-test effect size measures (applied in the first cycle only), (5) applying a smaller, 0%, (6) and a larger, 6%, discount rate to both costs and effects and (7) including only intervention completers in the analysis and (8) using a shorter time horizon of four years to reflect decision-makers mandate periods in Sweden.

## Results

**Fig 2:**
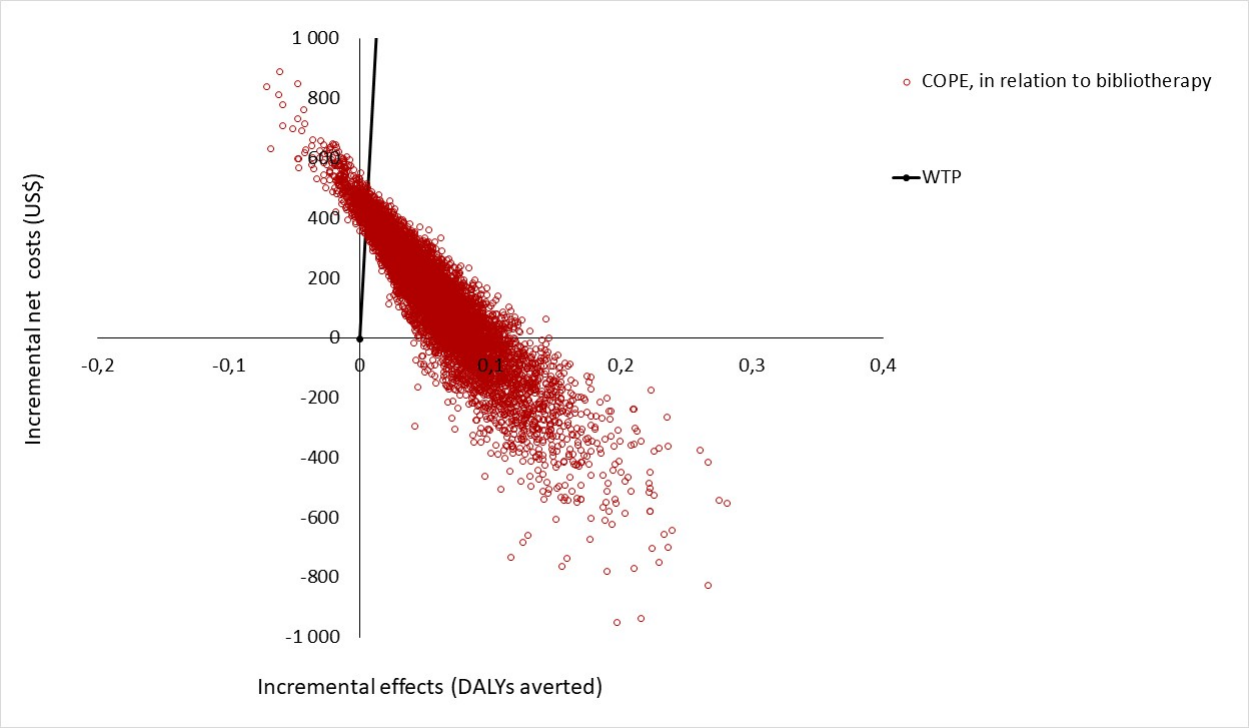
Cost effectiveness plane for base case analysis. Notes: DALYs = Disability-adjusted life-years.

Table 4 shows the results from the base case analyses of the cost-effectiveness simulations. Bibliotherapy had the lowest intervention cost of US$ 14 per child, whereas the IY had the highest (US$ 1,302). The IY had the highest effect in terms of averted DALYs, 0,23 per participant, while Connect had the lowest with 0,06 averted DALYs over the whole modeling horizon. Bibliotherapy, Connect and COPE *dominated* the comparator. This means both lower accumulated net costs (until the age of 18) and greater health benefits in terms of averted DALYs compared to the waitlist control. All parenting programs and bibliotherapy were 100% likely to be cost-effective at a US$ 80,000 WTP threshold per DALY compared to the waitlist control, and remained so when lowering the WTP to US$ 15,000. Results are also shown on a cost-effectiveness plane in Fig 2.

**Table 4:** Mean results (US$) of the base case cost-effectiveness model of four parenting programs and bibliotherapy, in comparison to the waitlist control.

							Incremental																								
Intervention	Intervention costs						Costs related to problems							DALYs averted						Net monetary benefit						ICER (US$/DALY averted)					
																			
Comet	931	(	926	-	935	)	790	(	779	-	802	)		0,14	(	0,14	-	0,15	)	7075	(	6932	-	7217	)	972	(	990	-	954	)
Connect	334	(	333	-	336	)	344	(	340	-	349	)		0,06	(	0,06	-	0,06	)	3097	(	3038	-	3156	)	dominant					
Incredible Years	1302	(	1296	-	1308	)	1250	(	1231	-	1269	)		0,23	(	0,23	-	0,24	)	11614	(	11373	-	11854	)	224	(	228	-	220	)
COPE	477	(	475	-	480	)	942	(	928	-	955	)		0,17	(	0,17	-	0,17	)	9029	(	8859	-	9199	)	dominant					
Bibliotherapy	14	(	14	-	14	)	617	(	608	-	626	)		0,11	(	0,11	-	0,12	)	6270	(	6155	-	6384	)	dominant					

Abbreviations: DALYs—Disability-adjusted life years, ICER—Incremental cost-effectiveness ratio. All results are presented as mean values per individual and with 95% confidence intervals. Results are presented in 2015 US$

The results from comparing each trial arm to one another through the use of extended dominance are shown in Table 5. When ranking the alternatives from the lowest to the highest net costs and comparing each intervention with the consecutive, bibliotherapy weakly dominated COPE, and COPE strongly dominated the rest. An ICER of US$ 2,629 was calculated for COPE in comparison to bibliotherapy.

**Table 5:** Determination and results from principle of extended dominance.

					Incremental					
Rank	Intervention	Net cost	Total DALYs	Comparison	Net cost	DALYs	Decision			
**Step #1**	** **	** **	** **	** **	** **	** **	** **			
1	Bibliotherapy	3 292	0,55							
2	COPE	3 432	0,49	1 vs. 2	- 140	0,06	weak dominance			
3	Connect	3 559	0,53	2 vs. 3	- 127	- 0,04	strong dominance			
4	Waitlist	3 918	0,66	2 vs. 4	- 486	- 0,17	strong dominance			
5	Comet	4 024	0,52	2 vs. 5	- 592	- 0,03	strong dominance			
6	IY	4 411	0,52	2 vs. 6	- 979	- 0,02	strong dominance			
**Step #2**							ICER*	NMB*	**Probability of cost-effectivenessᵃ**	
** **										
1	Bibliotherapy	3 292	0,55		- 140	0,06				
2	COPE	3 432	0,49		- 127	- 0,04	2629 (2552–2771)	2684 (2591–2778)	0,95	

Abbreviations: DALYs—Disability-adjusted life years, ICER—Incremental cost-effectiveness ratio, NMB—Net Monetary Benefit, net cost equals to the sum of the intervention cost and the average costs related to the prevalence of the problem states per individual

*ICER and NMB are derived from the probabilistic model with 1000 Monte Carlo simulations.

ᵃ Results are presented as mean values per individual (and with 95% confidence intervals for the final ICER and NMB)

Results are presented in 2015 US$

Net monetary benefits from eight sensitivity analyses are shown in Table 6 for each active intervention in relation to the waitlist, and for COPE versus bibliotherapy. In comparison to the base case model, the results varied marginally, except for the second and last analysis. In the second, the interventions’ effects were estimated based on both “recovered” and “improved” cases (detailed definitions can be found in the technical report [30]). The incremental net monetary benefits increased by up to 50% in some cases (Connect US$ 3,087 per child at base case to US$ 6,790). In the last analysis, where the time horizon was shortened to four years, the cost offsets dropped sharply, however the net monetary benefits remained positive. Overall, COPE and bibliotherapy dominated the waitlist control throughout the analyses. Throughout the analyses, the interventions were at least 90% likely to be cost-effective at a WTP threshold of US$ 80,000.

**Table 6:** Net monetary benefits (US$) of each univariate sensitivity analysis of the five interventions in relation to the waitlist, and for COPE versus Bibliotherapy.

Active interventions vs. Waitlist control																																				COPE vs. Bibliotherapy						
	Comet							Connect							IY							COPE							Bibliotherapy							COPE						
(1)	6832	(	6692	-	6971	)		2955	(	2897	-	3013	)		11259	(	11024	-	11495	)		8768	(	8599	-	8937	)		6120	(	6009	-	6230	)		2605	(	2512	-	2697	)	
(2)	10607	(	10406	-	10809	)		6790	(	6660	-	6920	)		12419	(	12162	-	12676	)		12295	(	12070	-	12520	)		7316	(	7183	-	7449	)		5020	(	4829	-	5212	)	
(3)	6151	(	6007	-	6294	)		2727	(	2669	-	2786	)		7798	(	7621	-	7975	)		8501	(	8332	-	8670	)		6253	(	6139	-	6367	)		2246	(	2126	-	2367	)	
(4)	4492	(	4399	-	4585	)		769	(	751	-	787	)		5672	(	5548	-	5796	)		4527	(	4441	-	4613	)		3364	(	3306	-	3423	)		2779	(	2659	-	2900	)	
(5)	7177	(	7034	-	7321	)		3141	(	3081	-	3201	)		11813	(	11572	-	12055	)		9125	(	8955	-	9296	)		6334	(	6219	-	6448	)		2696	(	2578	-	2814	)	
(6)	6987	(	6844	-	7129	)		3000	(	2942	-	3059	)		11373	(	11136	-	11610	)		8833	(	8667	-	9000	)		6189	(	6077	-	6300	)		1188	(	1084	-	1293	)	
(7)	6764	(	6621	-	6908	)		2753	(	2694	-	2812	)		7837	(	7659	-	8014	)		6212	(	6099	-	6326	)		6212	(	6099	-	6326	)		1915	(	1795	-	2036	)	
(8)	3575	(	3511	-	3639	)		1651	(	1622	-	1680	)		4263	(	4188	-	4338	)		4722	(	4650	-	4795	)		3431	(	3382	-	3480	)		2363	(	2265	-	2461	)	

Notes. (1) including only educational sector costs, (2) “recovered” and “improved” cases as effectiveness, (3) doubling intervention costs, (4) using only post-test effect size measure, (5) discount rate of 0, (6) discount rate of 6%, (7) only intervention completers, (8) time horizon of four years. All results are presented as mean values per individual with 95% confidence intervals in 2015 US$.

## Discussion

### Summary of findings

Using data from a randomized trial conducted in a “real-life” setting in Sweden, we estimated the longer-term cost-effectiveness of five parenting interventions and a passive waitlist control, for the prevention of persistent behaviour problems. Children aged 5–12 whose parents received an intervention to improve parenting management skills were followed until the age of 18 in a stochastic economic model. All the interventions were cost-effective at a threshold of US$ 80,000 per averted DALY, in comparison to the control group. By the principle of extended dominance, the additional cost for an extra averted DALY for COPE, in relation to bibliotherapy, was US$ 2,629. The results were robust through a series of sensitivity analyses that moderately changed the magnitude rather than the direction of the results.

### Comparison to other work

In relation to other studies looking at the cost-effectiveness of parenting interventions, the results follow a similar directional pattern, indicating that they are good *value for money*. Using within trial estimates and individual level data, Herman et al. [59] showed that the monetary benefits generated from the parenting intervention New Beginnings Program, 15-years after its implementation, were greater than the implementation costs when considering mental health services and criminal justice system costs. By applying secondary data for decision-analytic modelling purposes, a recent study has also shown that the Triple P parenting program, both delivered individually and in a group format, is cost-effective in the long-run for treating conduct disorders in Australia [25]. Bonin et al. [44] also used a decision-analytic model for a hypothetical parenting intervention for the prevention of persistent conduct disorder in the United Kingdom. She concluded that a parenting intervention may be cost-saving over a time period of 25 years to both the public sector and the society as a whole. O’Neill et al. [20] estimated an economic rate of return of 11% in the long-run by the IY program through its effect on education, crime and unemployment by reducing conduct problems in childhood.

### Policy and research implications

As both the EU health program and national agenda’s for public health directs increasing attention and funds towards mental health, prevention and sustainability [1], it is beneficial to consider the evidence portrayed of the longer term impact of parenting interventions. As a critical issue, the sector responsible for providing these interventions, such as the social services in Sweden, may not be the ones’ reaping the benefits. Discussion is therefore key to consider whether costs and benefits ought to be allocated proportionally to what is being gained by each sectors, and over different time periods. These type of longer-run analyses are also interesting from a sustainability perspective, and with a growing number of municipalities setting up social investment funds [60], there is an interest in estimating the economic benefits from early interventions. However, visualizing benefits through forecasts that stretch up to 13 years post-intervention might not be of practical use, as many decision makers have short mandate periods, usually four years in Sweden, and a pressure to produce economically beneficial results within that limited timeframe. Hence, short-term political constraints may stand in the way of long-term sustainability goals, whereby regardless of the discount rate on future benefits, the gains might still not be taken into account. However, as the results from the final sensitivity analysis showed, the net monetary benefits remained positive; thus, it is still economically attractive to allocate resources to these interventions. To look at the results more in detail, bibliotherapy is an inexpensive yet relatively effective option and may be easily disseminated to target behaviour problems. As a first line option under budget constraints, it is generally preferable in comparison to the face-to-face delivered programs. If decision-makers are willing to make larger investments, COPE produces higher effects. One may consider delivering parenting interventions in a stepped-care model of different formats and doses depending on demand and available funding. However, the effects of such delivery needs further investigation.

### Strengths and limitations

One of the major strengths of the current study is the possibility to compare several interventions within the same trial conducted in a “real-life” setting. This makes results more applicable to decision-making within public health, and may be relevant as economic evidence for priority setting. As the evaluated interventions are some of the most frequently used in Sweden, the head-to-head comparison of each intervention is of direct relevance. In addition, by using the statistical approach CS/RCI to estimate “recovered” cases, we are more likely to capture the individuals that have made a “real” improvement with an impact on costs. This is in relation to relying on cases/non-cases where many individuals could have made small changes around the clinical threshold, which would probably not lead to a discontinuation of using societal services.

As with previous studies within the area, the results are based on a limited costing perspective, which is likely to miss various societal impacts. Firstly, only health care and educational sector costs were included, and costs related to, for instance, the justice and voluntary sector, are missing due to lack of incremental cost data. In addition, costs are based on few studies, which makes cost estimated less robust. Secondly, parenting interventions are known to improve caregiver mental health [26], but related costs are not included in the analysis. If results are to be used for decision-making, a broader perspective is recommended [61]. In addition, the data used in the analyses are to a large extent based on Swedish estimates and the sample of help-seeking parents who participated in the trial. Four different starting points were used in the model as the interventions are targeted (thus included children both below and above clinical cut-offs), hence the baseline prevalence may differ from the average parenting program group. Results should therefore be generalized with caution to other populations. However, we believe that as the original trial was implemented within a real-life setting where participating units were responsible for recruitment according to their normal procedures, potential bias is reduced. In addition, we did not include all possible transitions between the health states in the model to limit the amount of assumptions, due to limited data availability to inform such transitions. For instance, no transition was directly possible between CP and ADHP/CP. This may have underestimated the proportion of individuals with problems as they might have been forced to remit in the model, rather than shifting to another problem state in reality. In addition, oppositional defiant disorder (ODD) was excluded from the model, although it is highly comorbid with the included problem states, as well as measured in the original trial. However, it was not possible to include ODD as a problem state because it is not measured in the GBD. This may have affected both the general epidemiology, how the interventions’ affect the problems and therefore the costs. However, the magnitude of such bias is difficult to estimate. It also remains important to consider, from an equity standpoint, who the beneficiaries of these interventions are [62]. Future work should collect enough data to perform sub-group analyses based on equity considerations, for instance to investigate whether socio-economic status, ethnicity etc. may mediate the cost-effectiveness results. Also, the health outcomes of children are based on parental-proxy rather than on self-report (as often done for estimating problem behaviours in children), which may have biased both the epidemiological and the effectiveness data. In addition, since there was no waitlist control after post-test measurement, we assumed that the proportion of recovered cases at post-test would be the same at follow-up for the waitlist. Other studies have shown conflicting results regarding sustainability of effects [16], thus it is unclear whether or not our assumption overestimates the findings. However, as seen in one of the sensitivity analyses, excluding the follow-up effect measures does not have a large impact on the results. The results are also based on aggregated effectiveness and cost data. No cost data was collected alongside the trial, hence cost proxies were estimated from the literature. Nevertheless, probabilistic and univariate sensitivity analyses were conducted to analyse the potential over/under-estimations of the cost data.

## Conclusion

The parenting programs evaluated in this study were cost-effective at a low willingness-to-pay threshold in relation to a waitlist control group. Bibliotherapy or COPE are the most efficient options when comparing interventions to one another, where bibliotherapy is the most inexpensive. However, if decision-makers are willing to invest more in return of higher effects on externalizing problems, the IY reduced the highest amount of DALYs. Results provide important evidence for sustainable investments in child health.
